# Brachial-ankle pulse wave velocity increasing with heart rate accelerates

**DOI:** 10.3389/fcvm.2023.1280966

**Published:** 2023-11-02

**Authors:** Qian Wang, Xinxin Xu, Xue Geng, Haijuan Hu, Wei Cui

**Affiliations:** ^1^ Department of Cardiovascular Medicine, The Second Hospital of Hebei Medical University, Shijiazhuang, China; ^2^ Department of Cardiovascular Medicine, Cangzhou People’s Hospital, Cangzhou, China

**Keywords:** pulse wave velocity, heart rate, blood pressure, arterial stiffness, pacemaker

## Abstract

Pulse wave velocity (PWV) indicates the degree of vascular stiffness. This study aimed to explore the association between heart rate (HR) and brachial-ankle (ba)-PWV in patients with pacemaker implantation. This retrospective observational study included patients who underwent permanent pacemaker implantation at the Second Hospital of Hebei Medical University between December 2018 and December 2021. All patients were pacemaker-dependent, and the ba-PWV values were collected during HR setted from 60 to 100 bpm. A total of 68 patients (34 males, aged 65.97 ± 9.90 years) were included in this study. There were significant difference of ba-PWV and diastolic blood pressure (DBP) among different HR (both *P* < 0.001). After adjusted systolic blood pressure (SBP), DBP, age, and sex, the generalized estimating equation showed ba-PWV was independently associated with HR, with increased HR showed higher coefficient: 70 bpm: *β* = 42.26 (95% CI: 15.34–69.18, *P* = 0.002), 80 bpm: *β *= 84.16 (95% CI: 52.48–115.84, *P* < 0.001), 90 bpm: *β* = 129.27 (95% CI: 52.48–115.84, *P* < 0.001), and 100 bpm: 186.31 (95% CI: 137.02–235.59, *P* < 0.001). The results demonstrate that changes in HR may affect the ba-PWV, the ba-PWV values tend to be higher when HR accelerates.

## Introduction

Pulse wave velocity (PWV) indicates the degree of vascular stiffness (1, 2). The PWV values also reflect the severity of atherosclerosis (2, 3). PWV correlates intimately with age (4), blood pressure (5), and diabetes (6, 7), and is a reliable tool for assessing cardiovascular (CV) risk (2, 8). Heart rate (HR) is probably the most widely used vital sign. An accelerated HR can contribute to the mechanical changes in the arteries induced by age and high blood pressure (BP) (9, 10). An accelerated HR is associated with CV and overall mortality (11, 12).

Technically, HR can influence PWV measurement and, consequently, its diagnostic or prognostic accuracy (13). Clinically, there may be a combined or synergistic effect of increased PWV and elevated resting HR on CV risk assessment (13). Still, whether HR would play an independent role in PWV variation remains controversial. Although previous studies reported PWV and HR were independently associated (14, 15), only a few studies reported that acutely increasing the HR leads to significant increases in PWV (15–17), probably due to the frequency-dependent viscoelasticity of the arterial walls (15). Only one study reported that an increased resting HR is related to increased aortic stiffness, regardless of BP (13). Therefore, this study aimed to explore the association between HR and brachial-ankle (ba)-PWV in patients with pacemaker implantation.

## Materials and methods

### Study design and patients

This retrospective observational study included patients who underwent permanent pacemaker implantation at the Second Hospital of Hebei Medical University between December 2018 and December 2021. The inclusion criteria were: (1) patients with bradycardia, (2) underwent pacemaker implantation, and (3) pacemaker-dependent. The exclusion criteria were: (1) refuse to accept the PWV examination or (2) ankle-brachial pressure index (ABI) < 0.9. The study was approved by the ethics committee of the Second Hospital of Hebei Medical University (approval #2021-R274). All patients provided written informed consent to PWV measurement.

### Data collection and definition

ba-PWV was measured using an automatic computerized technique (OMRON Healthcare, Kyoto, Japan). Using two mechanical transducers, pressure waves were recorded transcutaneously at two sites. Occlusion cuffs were tied around the upper arms and ankles while the patient was lying in the supine position. The ba-PWV was calculated as the distance between the two transducers and the time delay between the arm-to-foot pressure waves. Systolic BP (SBP) and diastolic BP (DBP) were measured simultaneously, and an average of the right and left arms measurements was taken for each patient as their reference BP. An average of two measurements were taken for each patient. In addition, the ABI was calculated bilaterally as the ratio of ankle-SBP to brachium-SBP on the left and right sides.

For each patient, the HR was increased at five steady-state HRs: 60, 70, 80, 90, and 100 bpm, and ba-PWV was determined at each HR simultaneously. As some patients may have a spontaneous sinus rhythm if the pacing is programmed too slow, 60 bpm was used as the slowest HR for this procedure. The upper limit of HR was 100 bpm to avoid any discomfort of the patients. An interval of at least 5 min was allotted between the HR changes and the measurements. It represents the most reasonable explanation for the results in that, because of the controlled experimental conditions, HR was almost the only parameter that seriatim varied. At the end of the study, the pacemaker was reprogrammed according to the patient's disease.

### Statistical analysis

PASS 15.0 Power Analysis and Sample Size Software (2017). (NCSS, LLC) was used for sample size calculation. The power analysis for the primary endpoint, which on determining the correlation between heart rate and ba-PWV, was calculated with a significance level of 5% based on a one-way repeated measures. SPSS 26.0 (IBM, Armonk, NY, USA) was used for statistical analysis. The continuous data were presented as mean ± standard deviation (SD). The categorical data were presented as *n* (%). One-way analysis of variance (ANOVA) for repeated measures was conducted to explore the association between ba-PWV, systolic BP (SBP), diastolic BP (DBP) and HR. And a generalized estimation equation (GEE) was used to analyze the association between ba-PWV and HR after adjusted for SBP, DBP, age, and sex.

Pearson's correlation analysis was conducted to analyze the correlation between HR and ba-PWV for each patient, and patients showed significant correlation were defined as correlated group, those showed no significant correlation were defined as non-correlated group. Two-way ANOVA for repeated measures were conducted to compare the ba-PWV, SBP, and DBP between the two groups. A two-sided *P* < 0.05 were considered as statistically significant.

## Results

### Characteristics of the patients

Based on our calculations using the PASS software, a sample size of 58 patients assigned to the pacemaker program and ba-PWV test would provide the study with 90% statistical power to detect the correlation between heart rate and ba-PWV. Considering an anticipated dropout rate of 10%–20%, it is reasonable to increase the sample size to 63–73. Therefore, a total of 68 patients were included in this study, the mean age was 65.97 ± 9.90 years, and there were 34 men and 34 women. The chronic diseases included obesity (4.41%), hypertension (52.94%), coronary heart disease (20.59%), diabetes (16.18%), and stroke (22.06%). The indications for pacing were atrioventricular block (*n* = 20, 29.41%) and sick sinus syndrome (*n* = 48, 70.59%). Five (7.35%) patients had only ventricular stimulation with atrial fibrillation, and 63 (92.65%) had dual-chamber stimulation. Thirty-six (52.94%) patients had hypertension, 14 (20.59%) had coronary heart disease, 13 (19.12%) had hyperlipidemia, 11 (16.18%) had diabetes, and 24(35.29%) were smokers. All patients had normal systolic and diastolic left ventricular function (Table 1).

**Table 1:** Demographic aynd clinical characteristics of the patients.

Characteristics	Mean ± SD or *n* (%)
Age (years)	65.97 ± 9.9
Sex, *n* (%)	
Male	34 (50.00)
Female	34 (50.00)
Obesity (BMI >30 kg/m^2^), *n* (%)	3 (4.41)
Hypertension, *n* (%)	36 (52.94)
Hyperlipidemia, *n* (%)	13 (19.12)
Coronary heart disease, *n* (%)	14 (20.59)
Diabetes, *n* (%)	11 (16.18)
Stroke, *n* (%)	15 (22.06)
Smoke, *n* (%)	24 (35.29)

SD, standard deviation; BMI, body mass index.

The one-way ANOVA for repeated measures showed that ba-PWV (*P* < 0.001) and DBP (*P* < 0.001) were significantly related to HR. Pairwise comparison showed significant differences of ba-PWV between every two adjacent HR (all *P* < 0.05). The associations between SBP and HR was not significant (*P* = 0.209) (Table 2).

**Table 2:** Results of the one-way analysis of variance for repeated measures.

Characteristics		F	*P*
ba-PWV (cm/s)			
HR (bpm)		38.12	<0.001
60	1,746.74 ± 50.90		
70	1,787.85 ± 53.87^a^		
80	1,844.43 ± 58.26^a^		
90	1,891.46 ± 62.88^a^		
100	1,947.02 ± 63.90^a^		
SBP (mmHg)			
HR (bpm)		1.512	0.209
60	138.75 ± 18.69		
70	137.85 ± 17.71		
80	138.37 ± 17.21		
90	137.49 ± 17.53		
100	136.60 ± 16.31		
DBP (mmHg)			
HR (bpm)		14.256	<0.001
60	79.40 ± 9.24		
70	80.13 ± 9.40		
80	80.75 ± 9.69		
90	82.23 ± 9.30^a^		
100	83.21 ± 9.28		

^a^ *P* < 0.05 compared to the previous HR.

ba-PWV, brachial-ankle pulse wave velocity; HR, heart rate; SBP, systolic blood pressure; DBP, diastolic blood pressure.

The GEE showed that after adjusted SBP, DBP, age, and sex, the association between ba-PWV and HR was significant, with increasing HR showed higher coefficient: 70 bpm: *β* = 42.26 (95% CI: 15.34–69.18, *P* = 0.002), 80 bpm: *β* = 84.16 (95% CI: 52.48–115.84, *P* < 0.001), 90 bpm: *β* = 129.27 (95% CI: 52.48–115.84, *P* < 0.001), and 100 bpm: *β* = 186.31 (95% CI: 137.02–235.59, *P* < 0.001) (Table 3).

**Table 3:** Generalized estimating equations of ba-PWV.

Parameters	*β*	Standard error	95% Wald confidence interval		Hypothesis testing		
			Lower limit	Upper limit	Wald *χ*^2^	*γ*	*P*
(intercept)	−1,358.34	442.27	−2,225.17	−491.52	9.43	1	0.002
[HR = 100]	186.31	25.15	137.02	235.59	54.89	1	<0.001
[HR = 90]	129.27	19.61	90.84	167.71	43.47	1	<0.001
[HR = 80]	84.16	16.16	52.48	115.84	27.12	1	<0.001
[HR = 70]	42.26	13.73	15.34	69.18	9.47	1	0.002
[HR = 60]	0^a^						
SBP	10.13	2.54	5.16	15.10	15.94	1	<0.001
DBP	8.95	5.62	−2.07	19.97	2.53	1	0.112
Age	14.57	3.45	7.81	21.32	17.84	1	<0.001
[Sex = male]	61.77	72.70	−80.73	204.27	0.72	1	0.396
[Sex = female]	0^a^						
(Scale)	1,20,276.22						

^a^ As this parameter is redundant, it is set to zero.

ba-PWV, Brachial-ankle pulse wave velocity; HR, heart rate; SBP, systolic blood pressure; DBP, diastolic blood pressure.

As each patient is an independent individual, correlation analyses were performed between ba-PWV and HR in each patient. There were significant correlations between ba-PWV and HR in 61 (89.71%) patients (all *P* < 0.05), and the correlations were not significant in 7 (10.29%) patients (all *P* > 0.05). The two-way ANOVA for repeated measures showed that there were no significant difference in ba-PWV, SBP, and DBP between the two groups (Supplementary Tables S1–S3). And the graphical result showed the ba-PWV, SBP, and DBP showed trends to be lower in non-correlated group (Supplementary Figures S1–S3).

## Discussion

This study suggested that the HR may affect the ba-PWV among patients with pacemaker, the ba-PWV tend to be higher when HR accelerates. The results might help refine the understanding of the relationship between HR and PWV and design strategies to optimize HR and BP control to improve CV risk management.

It is widely accepted that atherosclerosis plays an important role in the development of CV diseases and that the extent of atherosclerosis can be tested by PWV (2, 8). The PWV value can reflect the elasticity of the corresponding artery, including the carotid-radial artery PWV, the carotid-femoral artery PWV (cf-PWV), the femoral-tibial artery PWV, and the ba-PWV (18). The ba-PWV is a simple, noninvasive method that correlates well with arterial stiffness and aortic PWV obtained by invasive recordings, and the validity and reproducibility of ba-PWV measurements are considerably high (19–21). A cross-sectional population-based study showed that among healthy men aged 40–49, ba-PWV was positively correlated with both central and peripheral PWV (18). In addition, subjects with higher ba-PWV had a significantly higher mortality rate (22–26). ba-PWV is associated with increased carotid intima-media thickness (20). Patients with higher PWV, including ba-PWV, have a significantly higher incidence of stroke and cardiovascular diseases (2, 8, 27).

The velocity at which an arterial pulsation propagates to the periphery is the PWV, which is affected by the local vascular elasticity and the vascular functional status. Factors affecting vascular elasticity include age, sex, blood pressure, lipemia, glycemia, smoking, and obesity, among others (1–7). Hence, the patient factors (age and sex) and pathological conditions (hyperlipidemia, diabetes, obesity, smoking, and chronic kidney disease) can also reduce vascular elasticity by promoting the progression of atherosclerosis and affecting the results of the PWV measurements (1–7). However, the impact of HR on BP and PWV is still controversial (14–17). Some studies have shown that HR can independently influence PWV (15–17). Other studies showed that ba-PWV was positively correlated with mean BP and HR and that the reduction in PWV observed after atenolol could be explained by changes in BP and HR (19, 28, 29).

An accelerated HR may lead to increased PWV because accelerating HR may speed up blood circulation, increasing the mean systemic circulation pressure and peripheral resistance. PWV measurement becomes unreliable if the circulation is disturbed during pulse propagation (15). Thus, confirming that the circulation of the lower limb artery from the iliac to the tibial region is normal is critically important to ba-PWV measurement accuracy. The presence/absence of peripheral arterial disease (PAD) should be verified based on the ABI (21). PAD is suspected if the ABI on the left or the right side is ≤0.9. In view of this, patients with ABI <0.9 were excluded from the present study to minimize the influence of PAD on ba-PWV test results and increase the reliability of the results.

Bikia et al. (30) proposed “an in-silico evaluation” to interpret the impact of HR on PWV. They estimated the direct effect of HR on PWV independently of the concomitant BP variations. Their results showed that the BP-independent effect of HR on cf-PWV was estimated to be approximately 0.16 m/s per 10 bpm and 0.26 m/s per 10 bpm in cases with decreased and increased arterial stiffness, respectively. As a result, the study provided a strong and clinically relevant background for establishing cf-PWV correction for HR changes (30). Similarly, the present study on the relationship between ba-PWV and HR reached a similar conclusion. The present study showed a tendency to increase the ba-PWV levels within the same patient with faster HR. Therefore, it demonstrated that HR is an important factor in the individual variation of ba-PWV measurements. It also aligns with some evidence from a study showing a significant association between HR and PWV (31).

The possible reasons for the influence of HR on ba-PWV value could include that PWV values are determined by the physical and geometrical properties of its transmission medium, including arterial elasticity, luminal diameter, blood viscosity, and blood density. Generally, the geometrical properties of the artery and blood density are relatively stable. Therefore, the PWV value is influenced by arterial elasticity to a large extent. In physics, speed equals wavelength/time; an increase in wave frequency leads to an increase in wave speed while the wavelength remains constant. Therefore, HR acceleration (i.e., the cardiac contraction frequency) causes an increase in PWV when the vessel's length remains constant (17). Because of the rapid HR changes, the neurohumoral status remains unchanged. Nakao et al. (32) suggested that ba-PWV is closely associated with sympathetic nervous activity in young Japanese men (32). Haesler et al. (33) showed that sympathetic activity did not increase with increasing HR. Another result is consistent with the present one in that the increase in PWV with increasing HR was independent of sympathetic activity since it occurred in intact as well as in sympathectomized rats (34). Since the effect of HR variation on PWV within individuals cannot be dismissed, HR should be considered when examining PWV changes.

It should be emphasized that all patients with pacemakers had normal systolic and diastolic left ventricular function. Therefore, the changes in ba-PWV induced by HR acceleration might have a minor correlation with the changes in the left ventricular contractile function and hemodynamics. Furthermore, all sets of measurements were performed within each patient, and each patient was controlled, increasing power.

Nevertheless, this study had limitations. It was performed at a single center, and the sample was small. Evaluating many more patients will be necessary to determine correction factors for ba-PWV in relation to HR. Large-scale longitudinal studies could also examine the impact of HR and ba-PWV on CV outcomes.

In conclusion, HR may affect the measurement of ba-PWV, and the ba-PWV tend to be higher when HR accelerates. HR is an important factor that can affect ba-PWV measurements. The effect of HR variation on PWV measurements within individuals cannot be dismissed. Further studies are needed to expound precisely what kind of correction should be applied to minimize this HR effect on PWV.

## Data Availability

The original contributions presented in the study are included in the article/**Supplementary Material**, further inquiries can be directed to the corresponding author.

## Supplementary material

Supplementary Figure S1 Trends of brachial-ankle pulse wave velocity (ba-PWV) and heart rate. Group 1: the correlated group; Group 2: the non-correlated group. (**A**). The ba-PWV of different heart rates between the two groups; (**B**). The ba-PWV of the two groups among different heart rates.

Supplementary Figure S2 Trends of systolic blood pressure (SBP) and heart rate. Group 1: the correlated group; Group 2: the non-correlated group. (**A**). The SBP of different heart rates between the two groups; (**B**). The SBP of the two groups among different heart rates.

Supplementary Figure S3 Trends of diastolic blood pressure (DBP) and heart rate. Group 1: the correlated group; Group 2: the non-correlated group. (**A**). The DBP of different heart rates between the two groups; (**B**). The DBP of the two groups among different heart rates.
