# Supplementary material for: Brachial-ankle pulse wave velocity increasing with heart rate accelerates
Source: Front Cardiovasc Med. 2023 Nov 2;10:1280966. doi: 10.3389/fcvm.2023.1280966 (PMC10652409; doi:10.3389/fcvm.2023.1280966)
Supplement: Supplementary file 1 [file Table1.docx]

**[Supplementary](javascript:;) Table S1.** Results of the two-factor analysis of variance of repeated measures of ba-PWV of patients with increasing heart rate (from 60 to 100 bpm)

| Group | Ba-PWV (cm/s) | F test | | |
| --- | --- | --- | --- | --- |
|  |  | F | P | Partial η^2^ |
| Correlated group | 1875.23±60.16 | 2.702 | 0.105 | 0.039 |
| Non-correlated group | 1567.01±177.58 |  |  |  |

[**Supplementary**](javascript:;) **Table S2.** Results of analysis of variance with two-factor repeated measures of SBP of patients with increasing heart rate (from 60 to 100 bpm)

| Group | SBP (mmHg) | F test | | |
| --- | --- | --- | --- | --- |
|  |  | F | P | Partial η^2^ |
| Correlated group | 138.90±2.13 | 2.532 | 0.116 | 0.037 |
| Non-correlated group | 128.36±6.27 |  |  |  |

[**Supplementary**](javascript:;) **Table S3.** Results of analysis of variance with two-factor repeated measures of DBP of patients with increasing heart rate (from 60 to 100 bpm)

| Group | DBP (mmHg) | F test | | |
| --- | --- | --- | --- | --- |
|  |  | F | P | Partial η^2^ |
| Correlated group | 81.45±1.14 | 0.703 | 0.405 | 0.011 |
| Non-correlated group | 78.47±3.36 |  |  |  |
