# Supplementary figures and images for: Brachial-ankle pulse wave velocity increasing with heart rate accelerates
Source: Front Cardiovasc Med. 2023 Nov 2;10:1280966. doi: 10.3389/fcvm.2023.1280966 (PMC10652409; doi:10.3389/fcvm.2023.1280966)

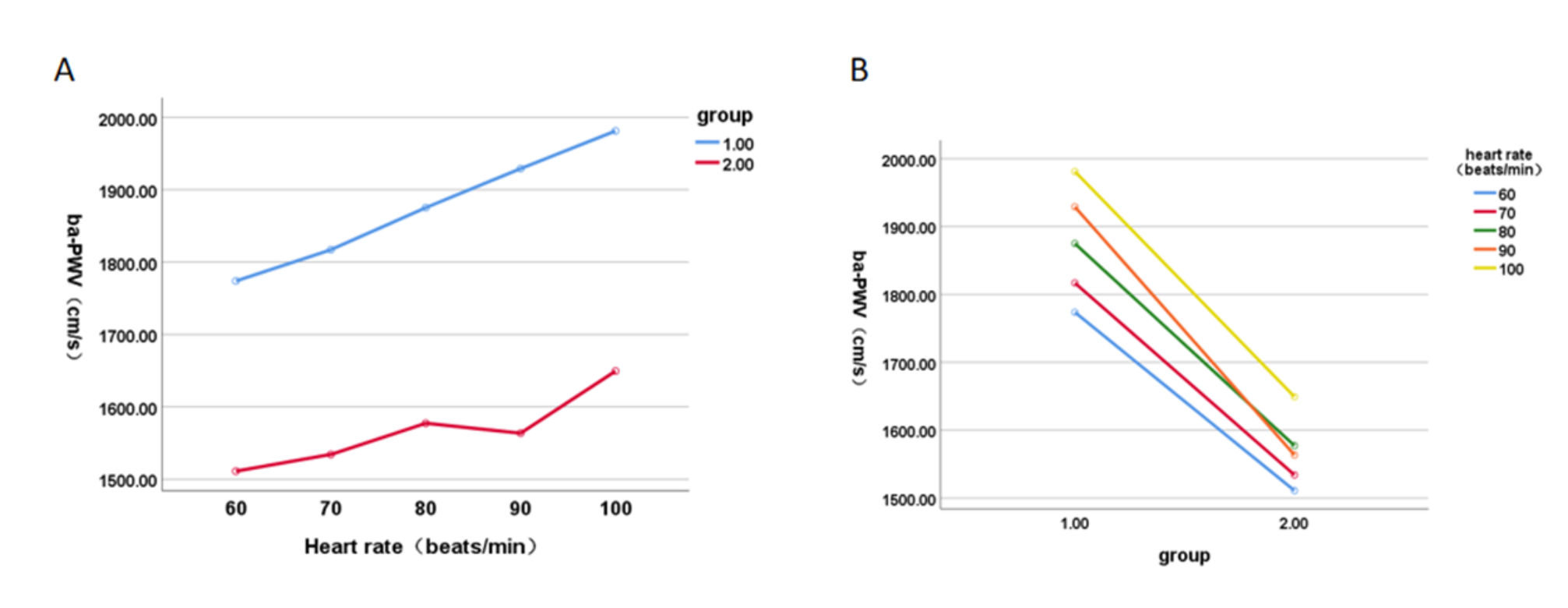

Supplement: Supplementary Figure S1 — Trends of brachial-ankle pulse wave velocity (ba-PWV) and heart rate. Group 1: the correlated group; Group 2: the non-correlated group. (A). The ba-PWV of different heart rates between the two groups; (B). The ba-PWV of the two groups among different heart rates. [file Image1.tiff]

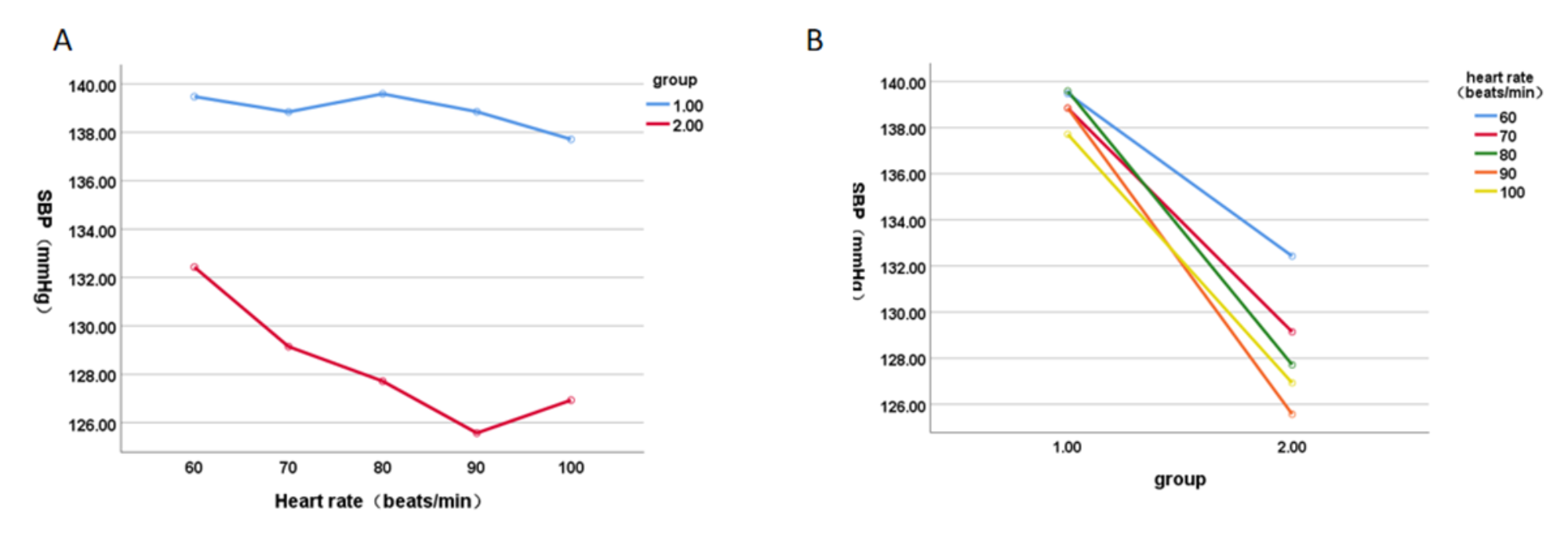

Supplement: Supplementary Figure S2 — Trends of systolic blood pressure (SBP) and heart rate. Group 1: the correlated group; Group 2: the non-correlated group. (A). The SBP of different heart rates between the two groups; (B). The SBP of the two groups among different heart rates. [file Image2.tiff]

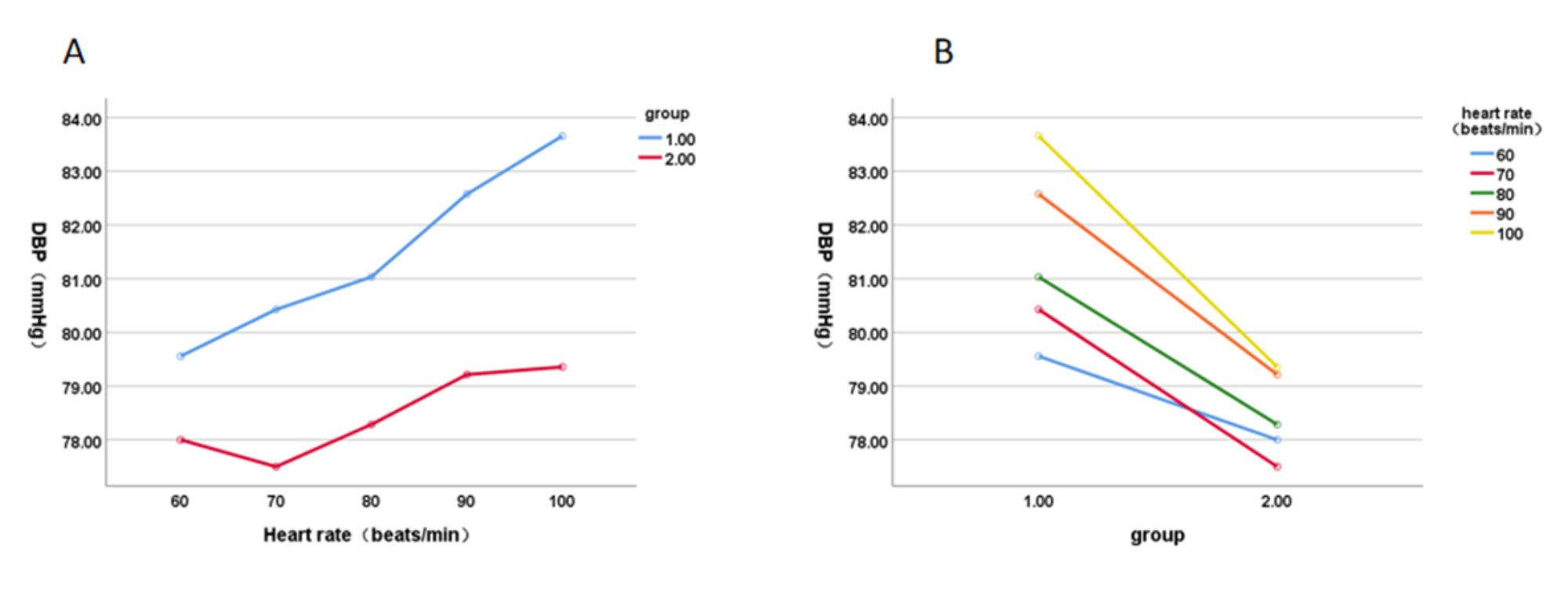

Supplement: Supplementary Figure S3 — Trends of diastolic blood pressure (DBP) and heart rate. Group 1: the correlated group; Group 2: the non-correlated group. (A). The DBP of different heart rates between the two groups; (B). The DBP of the two groups among different heart rates. [file Image3.tiff]
